# A reconfigurable multi-terrain adaptive casualty transport aid base on Watt II six-bar linkage for industrial environment

**DOI:** 10.3389/fbioe.2024.1360902

**Published:** 2024-03-28

**Authors:** Zongqi Jiao, Haibin Wang, Cuizhi Fei, Liang Wang, Jincan Yuan, Qiaoling Meng, Xuhua Lu

**Affiliations:** ^1^ Institute of Rehabilitation Engineering and Technology, University of Shanghai for Science and Technology, Shanghai, China; ^2^ Department of Orthopaedic Surgery, Changzheng Hospital, Naval Medical University, Shanghai, China

**Keywords:** casualty transport aid, Watt II six-bar linkage, reconfigurable robot, kinematics and statics, parametric study

## Abstract

**Introduction:** This paper presents the Reconfigurable Multi-Terrain Adaptive Casualty Transport Aid (RMTACTA), an innovative solution addressing the critical need for rapid and safe pre-hospital casualty transport in industrial environments. The RMTACTA, leveraging the Watt II six-bar linkage, offers enhanced adaptability through six modes of motion, overcoming the limitations of traditional stretchers and stretcher vehicles by facilitating navigation across narrow and challenging terrains.

**Methods:** The RMTACTA's design incorporates two branching four-bar mechanisms to form a compact, reconfigurable Watt II six-bar linkage mechanism. This setup is controlled via a single remote rope, allowing for easy transition between its multiple operational modes, including stretcher, stretcher vehicle, folding, gangway-passing, obstacle-crossing, and upright modes. The mechanical design and kinematics of this innovative linkage are detailed, alongside an analysis of the optimal design and mechanical evaluation of rope control.

**Results:** A prototype of the RMTACTA was developed, embodying the proposed mechanical and kinematic solutions. Preliminary tests were conducted to verify the prototype's feasibility and operability across different terrains, demonstrating its capability to safely and efficiently transport casualties.

**Discussion:** The development of the proposed Reconfigurable Multi-Terrain Adaptive Casualty Transport Aid (RMTACTA) introduces a novel perspective on the design of emergency medical transport robots and the enhancement of casualty evacuation strategies. Its innovative application of the Watt II six-bar linkage mechanism not only showcases the RMTACTA's versatility across varied terrains but also illuminates its potential utility in critical scenarios such as earthquake relief, maritime rescue, and battlefield medical support.

## 1 Introduction

Pre-hospital casualty transport is particularly critical for injured casualties suffering from limb and spinal fractures, brain injuries and visceral injuries (Wilson et al., 2015). Rapid and safe casualty transfer will allow the injured to receive care as soon as possible, enhancing survival and post-operative recovery rates (Bricknell, 2003; Cao et al., 2023; Yang et al., 2023). The complexity of casualty transfer is increased by the presence of narrow passageways, turns, gangways, and other obstacles in industrial environments like factories and ships (Butler FK et al., 2022). A fast and efficient casualty transport aid can help rescuers overcome these challenges and ensure that the casualty reaches the medical facility without incident. Stretchers and stretcher trucks are the most common casualty transport aid. The stretcher is frequently employed in field ambulances and disaster rescue due to its lightweight, portable structure and excellent environmental compatibility (Lim and Ng, 2021). However, there are safety risks associated with using the stretcher, including as overturning, a heavy load on the rescue team, a poor transfer pace, and other issues (Drobinsky et al., 2020). Stretcher vehicles, such as the Stryker Stretcher Vehicle, can provide more protection for the injured while also increasing transfer efficiency by switching from lifting to pushing. However, the stretcher vehicles’ larger size and heavier weight make them more suitable for more spacious areas such as hospitals and neighborhoods, making it difficult to pass easily through narrow environments and terrain with a significant difference in height, such as gangways and high thresholds (Qin and Li, 2023).

Advancements and innovations in casualty transfer aid have yielded significant results. Several researchers, including Xixi Hong (Hong et al., 2020), Lingfeng Sang (Sang et al., 2019), Aguilar-Pérez L A (Aguilar-Pérez et al., 2021) and Jin Rui (Rui and Gao, 2019), have developed equipment based on a multi-link design. This design allows for a seamless transition between a stretcher and a wheelchair, significantly reducing the equipment’s turning radius. As a result, it simplifies navigation in confined spaces such as elevators and tight corners. However, changing the stance of the stretcher and wheelchair will cause the casualty’s stance to alter, which is undesirable for badly injured people, such as those with spinal injuries. To tackle the difficulty of moving the victim through the stairs, transfer equipment with a range of auxiliary modules such as tracks (Iwano et al., 2011), active gimbals (Verjans et al., 2021a; Verjans et al., 2021b), passive gimbals (Feng et al., 2022), and so on has been devised for stairway terrain. The above research, however, is prone to uncontrolled falls and other problems when utilized on gangways in factories, ships, and other situations where gangways have greater slopes of up to 70° (community stairways have slopes of only 30°).

**FIGURE 1:**
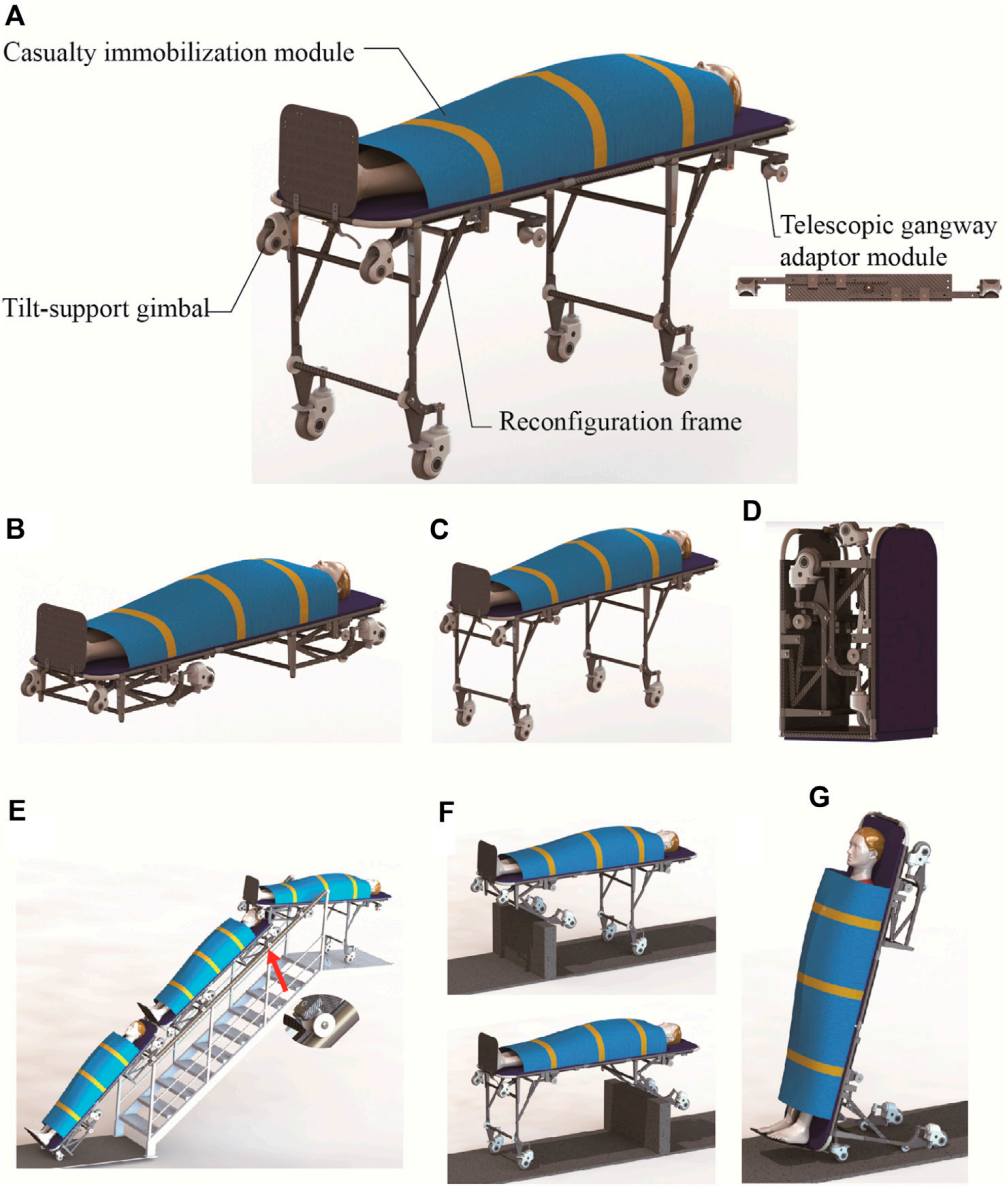
Mechanical structure of the RMTACTA and the six modes. **(A)** Mechanical structure of the RMTACTA. **(B)** Stretcher mode. **(C)** Stretcher vehicle mode. **(D)** Folding mode. **(E)** Gangway-passing mode. **(F)** Obstacle-crossing mode. **(G)** Upright mode.

**FIGURE 2:**
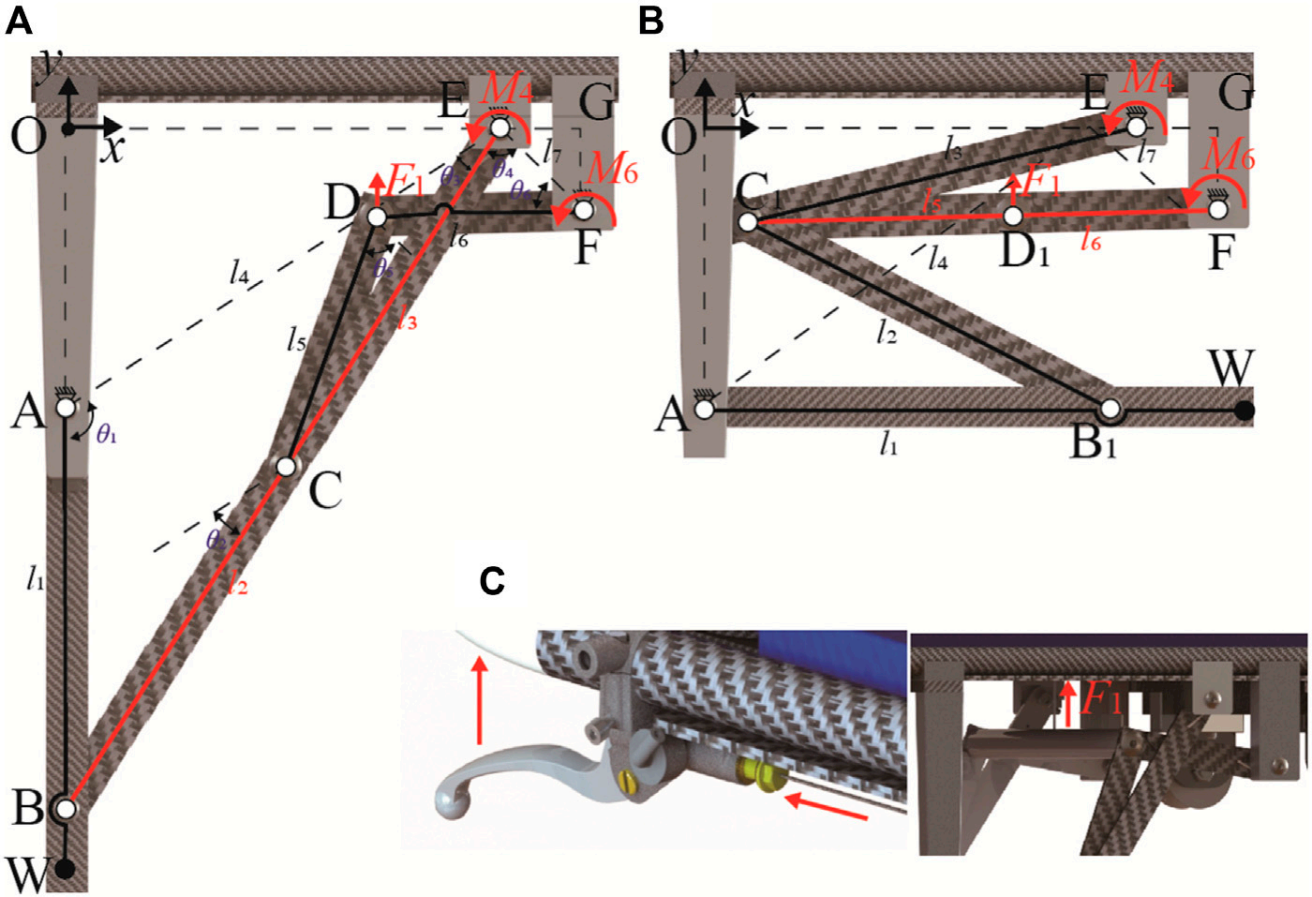
Geometry of the Watt II six-bar linkage in the reconfigurable frame. **(A)** Expansion mode. **(B)** Retracted mode. **(C)** Remote cable-driven mechanism.

Inspired by the aforementioned casualty transport aid and the analysis of industrial environment, this paper proposes a reconfigurable multi-terrain adaptive casualty transport aid (RMTACTA) with multiple motion modes as shown in Figure 1. The proposed RMTACTA possesses six different operating modes in total, including stretcher mode, stretcher vehicle mode, folding mode, gangway-passing mode, obstacle-crossing mode, and upright mode. This multipurpose tool can move across any kind of tight terrain quickly and safely, ensuring the relative calm of the victim and lightening the rescuer’s load. The reconfigurable frame of the proposed RMTACTA is formed by two branching four-bar mechanisms to form a Watt II six-bar linkage mechanism unit, which is manually controlled by only one remote rope, as shown in Figure 2C. The structure of the proposed casualty transfer robot is much more compact compared to the stretcher truck. Although the Watt II six-bar linkage has been widely used in various engineering applications such as rehabilitation exoskeletons, robotics, etc., this is the first time that it has been developed due to the reconfigurable casualty transport aid. The proposed RMTACTA, which is built on this reconfigurable frame, has numerous capabilities while still ensuring a compact structure. Although the Watt II six-bar linkage has been widely used in various engineering applications (Erdman et al., 2001; Waldorn et al., 2016) such as exoskeleton (Hyun et al., 2019), rehabilitation robot (Gezgin et al., 2016), mobile robot (Luo et al., 2018), etc., this is the first time that it has been developed due to the reconfigurable casualty transport aid.

The mechanical design of the RMTACTA is introduced and the kinematics of the reconfigurable Watt II six-bar linkage is presented. The kinematic mechanism of the Watt II six-bar linkage is revealed, and the optimal design of the linkage and the mechanical analysis of the rope control are performed. A prototype of the proposed RMTACTA is developed leading to the tests verifying its feasibility and operability.

## 2 Mechanical design of a reconfigurable multi-terrain adaptive casualty transport aid

The reconfigurable multi-terrain adaptive casualty transport aid (RMTACTA) proposed in this paper is a modular multi-locomotion transport aid, as shown in Figure 1A. It consists of two pairs of reconfigurable frames, two sets of telescopic gangway adaptor modules, a casualty immobilization module, and a pair of title-support gimbal.

The aid is designed for portability and can be compactly folded for storage in a rucksack, facilitating rapid transportation to emergency scenes (Figure 1D). It can be swiftly deployed into stretcher mode, facilitating the process of moving a casualty from the ground onto the stretcher and administering first aid (Figure 1B). For enhanced transfer speed on flat terrain, the aid transitions to stretcher trolley mode (Figure 1C). In the presence of a gangway, the device adapts to gangway passing mode. This is achieved by extending the pulleys of the telescopic gangway adaptor module, which engage with the gangway handrail, allowing smooth maneuvering of the equipment through the gangway (Figure 1E). Upon encountering obstacles such as high thresholds, the aid shifts to obstacle-crossing mode. This involves unlocking the reconfigurable frame, rotating the leg frame upon contact with the obstacle, and allowing it to automatically unfold and lock due to gravity. This feature enables the aid to traverse obstacles while ensuring continuous ground contact with a set of support wheels, thereby reducing the physical strain on the rescuer and maintaining the aid’s stability (Figure 1F). In scenarios involving elevators and tight turns, the aid converts to upright mode through the coordinated action of the reconfigurable frame and tilt-support gimbals. In this configuration, the tilt-support gimbals and leg frame wheels form a stable mobile platform, enabling the rescuer to navigate the aid through confined spaces with a minimal turning radius (Figure 1G).

The multifunctional motion modes of this aid are achieved through an innovative reconfigurable system, which includes a wire drive system and symmetrical reconfigurable frames. Each frame utilizes a Watt II six-bar linkage with two double-loop, four-bar configuration. This design employs the dead point states of the two four-bars to lock the wheel leg in both extended and retracted position. Torsion springs maintain the stability of the four-bars at the dead point positions, with the wheel legs serving as the output. The control system for each reconfigurable frame is wire-driven remote control system consisting of a Bowden cable and a handbrake located at the handle, which is used to provide actuation for disengaging the Watt II six-bar linkage from its two dead point states. This reconfigurable frame, in conjunction with the wire-driven remote control system, enables dual-mode switching via a single manual switch. Apart from the operation of extracting the pulleys to align with the gangway, all other functions can be controlled by the rescuer using a single handbrake, and throughout the process, both hands need not leave the device handle. This significantly reduces the operations required by the rescuer, which is of great importance in rescue situations (NAEMT, 2016).

## 3 Kinematics of Watt II six-bar linkage and configurations of the transport aid

Based on the mechanical design previously mentioned, it can be concluded that the multimodal transformation of the aid is realized through of the reconfigurable frame which is constructed using a Watt II six-bar linkage. In this section, the kinematics of the linkage is investigated, and the configuration of the structure is characterized, in order to expose the relationship between the configuration of the reconfigurable frame and the motion of the transport aid.

### 3.1 Kinematics of the Watt Ⅱ six-bar linkage

The schematic diagram of the Watt II six-bar linkage of the reconfigurable frame is given in Figure 2. Where, link AEF is a ternary V-shaped link, and the rest of links are the binary linkages. By using the V-shaped link as a fixed link that is solidly attached to aid’s body, a Cartesian coordinate system is established with the origin at point O, which the *y*-axis being isotropic to the vector AO, and *x*-axis being isotropic to the vector OE. The geometric parameters are further defined as: (1) For the branching four-link ABCE, AE is the fixed link, and *l*_1_, *l*_2_, *l*_3_, and *l*_4_ refer to the lengths of the links of AB, BC, CE, and AE (2) For the branching four-link ECDF, EF is the fixed link, and *l*_3_, *l*_5_, *l*_6_, and *l*_7_ refer to the lengths of the linkage of CE, CD, DF, and EF. (3) The joint angles *θ*_1_ to *θ*_6_ are defined as the linkage pinch angles, where *θ*_2_ is the angle between the parallel lines of BC and AD, and *θ*_5_ is the angle between the parallel lines of CD and EF for the convenience of kinematic modeling. (4) The input angle of the linkage is *θ*_1_ varying from 180-∠OAE to 90-∠OAE. (5) The Degree of Freedom (DoF) of the mechanism is calculated as F = 3N-2F_L_-F_H_ = 3×5–2×7 = 1.

During the mode switching process, the motion and locking of the wheel legs predominantly rely on the dead center position of the linkage mechanism, at which point the DoF of the mechanism is F = 3N-2FL-FH = 3×4–2×6 = 0. Consequently, this section investigates the displacements at points C and D, based on the geometric structure of the Watt II six-bar linkage. By decomposing the mechanism, the Watt II six-bar linkage is categorized into two sub-branches of four-bar linkages, namely, ABCE and ECDF. The displacement of point C is determined within the linkage ABCE, while the displacement of point D is deduced within the linkage ECDF.

In four-bar linkage ABCE based on loop equation (Michael McCarthy, 2010), it has

$$\left[\begin{array}{cc} \cos\theta_{1} & \cos\theta_{2}\\ \sin\theta_{1} & \sin\theta_{2} \end{array}\right]\left[\begin{array}{c} l_{1}\\ l_{2} \end{array}\right]=\left[\begin{array}{cc} \cos\theta_{3} & \cos\theta_{\text{AE}}\\ \sin\theta_{3} & \sin\theta_{\text{AE}} \end{array}\right]\left[\begin{array}{c} l_{3}\\ l_{4} \end{array}\right]$$ (1)

where *θ*_1_ is the input angle, the angle *θ*_AE_ of the fixed link AE is defined as 0° for simplicity of calculation and *l*_3_ = *l*_BE_-*l*_2_ in Figure 2A.

Solving Eq. 1 leads to the two variables, i.e., angles *θ*_2_ and *θ*_3_ as defined in Eq. 2.

$$\begin{array}{l} \theta_{2}=2\times \arctan\left(\left(-\beta -\sqrt{\beta^{2}-4\times \delta \times \varepsilon }\right)/\left(2\times \delta \right)\right)\\ \theta_{3}=2\times \arctan\left(\left(-\beta -\sqrt{\beta^{2}-4\times \alpha \times \gamma }\right)/\left(2\times \alpha \right)\right) \end{array}$$ (2)

where the expression for α, γ, β, δ, and ε are shown as

$$\begin{array}{l} \alpha =-l_{4}/l_{1}+\left(1-l_{4}/l_{3}\right)\times \cos\left(\theta_{1}\right)+\left({l_{4}}^{2}+{l_{1}}^{2}-{l_{2}}^{2}+{l_{3}}^{2}\right)/\left(2\times l_{1}\times l_{3}\right)\\ \beta =-2\times \sin\left(\theta_{1}\right)\\ \gamma =l_{4}/l_{1}-\left(1+l_{4}/l_{3}\right)\times \cos\left(\theta_{1}\right)+\left({l_{4}}^{2}+{l_{1}}^{2}-{l_{2}}^{2}+{l_{3}}^{2}\right)/\left(2\times l_{1}\times l_{3}\right)\\ \delta =-l_{4}/l_{1}+\left(1+l_{4}/l_{2}\right)\times \cos\left(\theta_{1}\right)+\left(-{l_{4}}^{2}-{l_{1}}^{2}-{l_{2}}^{2}+{l_{3}}^{2}\right)/\left(2\times l_{1}\times l_{2}\right)\\ \varepsilon =l_{4}/l_{1}-\left(1-l_{4}/l_{2}\right)\times \cos\left(\theta_{1}\right)+\left(-{l_{4}}^{2}-{l_{1}}^{2}-{l_{2}}^{2}+{l_{3}}^{2}\right)/\left(2\times l_{1}\times l_{2}\right) \end{array}$$

Thus, position of point C can be presented as

$$P_{C}=\left[\begin{array}{c} x_{C}\\ y_{C} \end{array}\right]=\left[\begin{array}{cc} x_{E} & \cos\left(\theta_{3}-\theta_{OEA}\right)\\ y_{E} & \sin\left(\theta_{OEA}-\theta_{3}\right) \end{array}\right]\left[\begin{array}{c} 1\\ l_{3} \end{array}\right]$$ (3)

Similarly, in the sub-branch four-bar linkage ECDF, position of point D can be deduced as

$$P_{D}=\left[\begin{array}{c} x_{D}\\ y_{D} \end{array}\right]=\left[\begin{array}{cc} x_{F} & \sin\left(\theta_{GFE}-\theta_{6}\right)\\ y_{F} & -\cos\left(\theta_{GFE}-\theta_{6}\right) \end{array}\right]\left[\begin{array}{c} 1\\ l_{6} \end{array}\right]$$ (4)

where *l*_6_ = *l*_C1F_- *l*_5_ in Figure 2B, and using loop equation it has

$$\theta_{6}=2\times \arctan\left(\left(-\beta^{\prime }-\sqrt{\beta \prime^{2}-4\times \alpha \prime \times \chi \prime }\right)/\left(2\times \alpha^{\prime }\right)\right)$$ (5)

where the expression for *α*, *γ*, *β*, *δ*, and ε are shown as

$$\begin{array}{l} \theta_{4}=180-\theta_{3}-\theta_{OEA}-\theta_{GEF}\\ \alpha^{\prime }=-l_{7}/l_{3}+\left(1-l_{7}/l_{6}\right)\times \cos\left(\theta_{4}\right)+\left({l_{7}}^{2}+{l_{3}}^{2}-{l_{5}}^{2}+{l_{6}}^{2}\right)/\left(2\times l_{3}\times l_{6}\right)\\ \beta^{\prime }=-2\times \sin\left(\theta_{4}\right)\\ \chi^{\prime }=l_{7}/l_{3}-\left(1+l_{7}/l_{6}\right)\times \cos\left(\theta_{4}\right)+\left({l_{7}}^{2}+{l_{3}}^{2}-{l_{5}}^{2}+{l_{6}}^{2}\right)/\left(2\times l_{3}\times l_{6}\right)\\ \delta^{\prime }=-l_{7}/l_{3}+\left(1+l_{7}/l_{5}\right)\times \cos\left(\theta_{4}\right)+\left(-{l_{7}}^{2}-{l_{3}}^{2}-{l_{5}}^{2}+{l_{6}}^{2}\right)/\left(2\times l_{3}\times l_{5}\right)\\ \varepsilon^{\prime }=l_{7}/l_{3}-\left(1-l_{7}/l_{5}\right)\times \cos\left(\theta_{4}\right)+\left(-{l_{7}}^{2}-{l_{3}}^{2}-{l_{5}}^{2}+{l_{6}}^{2}\right)/\left(2\times l_{3}\times l_{5}\right) \end{array}$$

In addition, angle *θ*_5_ can be obtained as

$$\theta_{5}=2\times \arctan\left(\left(-\beta \prime -\sqrt{\beta \prime^{2}-4\times \delta^{\prime }\times \varepsilon }\prime \right)/\left(2\times \delta^{\prime }\right)\right)$$ (6)

To guarantee that the reconfigurable mechanism achieves two locking positions, the structural parameters of the dual sub-branch four-linkages must fulfill the constraints: *l*_B1E_≤|*l*_3_-*l*_2_| and *l*_C1F_≤|*l*_5_-*l*_6_|.

### 3.2 Configurations and locomotion modes of the casualty transport aid

**FIGURE 3:**
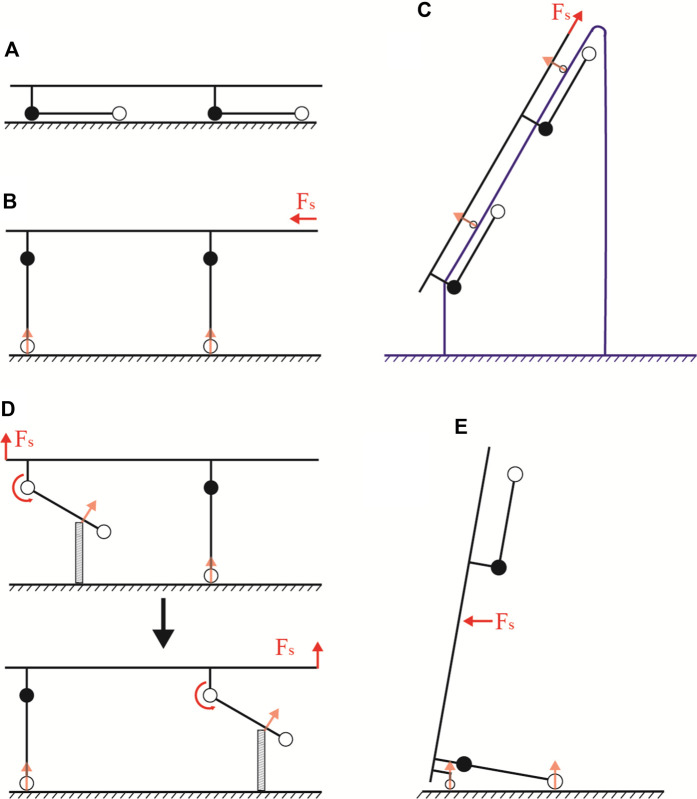
Locomotion modes of the transport aid. **(A)** Stretcher mode. **(B)** Stretcher vehicle mode. **(C)** Gangway-passing mode. **(D)** Obstacle-crossing mode. **(E)** Upright mode.

The reconfiguration of the Watt II six-bar linkage permits the wheel legs to lock in both the extended and retracted positions, forming the stretcher mode and the stretcher vehicle mode, respectively (refer to Figures 2A, B). This reconfiguration enables the aid to operate in five modes: stretcher mode, stretcher vehicle mode, obstacle-crossing mode, gangway-passing mode, and upright mode, as depicted in Figure 3.

The stretcher mode is primarily used for transporting casualty short distances from the ground to the stretcher (Figure 3A). The stretcher vehicle mode is particularly suited for rapid movement on flat terrain (Figure 3B). Upon encountering an obstacle, the Watt II six-bar linkage is unlocked, allowing the wheel legs to move freely. This transition transforms the stretcher vehicle mode into the obstacle-crossing mode (Figure 3D). In this mode, the aid ensures the casualty remains horizontal while crossing obstacles and maintains at least one set of support wheels on the ground, significantly reducing the rescuers’ effort and enhancing the aid’s stability.

Moreover, the stretcher mode, when combined with retractable pulleys, can be transformed into the gangway-passing mode for navigating through gangways (Figure 3C). Similarly, integrating tail casters with the stretcher vehicle mode enables the conversion to upright mode, facilitating maneuvering through narrow turns, elevators, and varied terrain, as shown in (Figure 3E).

## 4 Numerical simulation and parametric study

### 4.1 Numerical simulation and position synchronization

In order to validate the operational principle of the reconfigurable mechanism, numerical simulations based on the structural parameters outlined in Table 1 in this section. Here, *l*_OE_, *l*_EG_, *l*_GF_, *l*_1_, *l*_2_, *l*_5_, and *l*_6_ are identified as design variables, while the remaining parameters are determined through equation-based calculations. For clarity, the output angle of the reconfigurable mechanism, transitioning from retracted to extended, is defined as the angular variation when AB shifts from being parallel to the *x*-axis to parallel to the *y*-axis, and is established as ranging from 0 to 90°.

**TABLE 1:** Structure parameters of the reconfigurable frame in initial design.

Structure parameter	*l*_OA_	*l*_OE_	*l*_EG_	*l*_GF_	*l*_1_	*l*_2_	*l*_5_
Length(mm)	150	200	50	0	150	180.3	115

**FIGURE 4:**
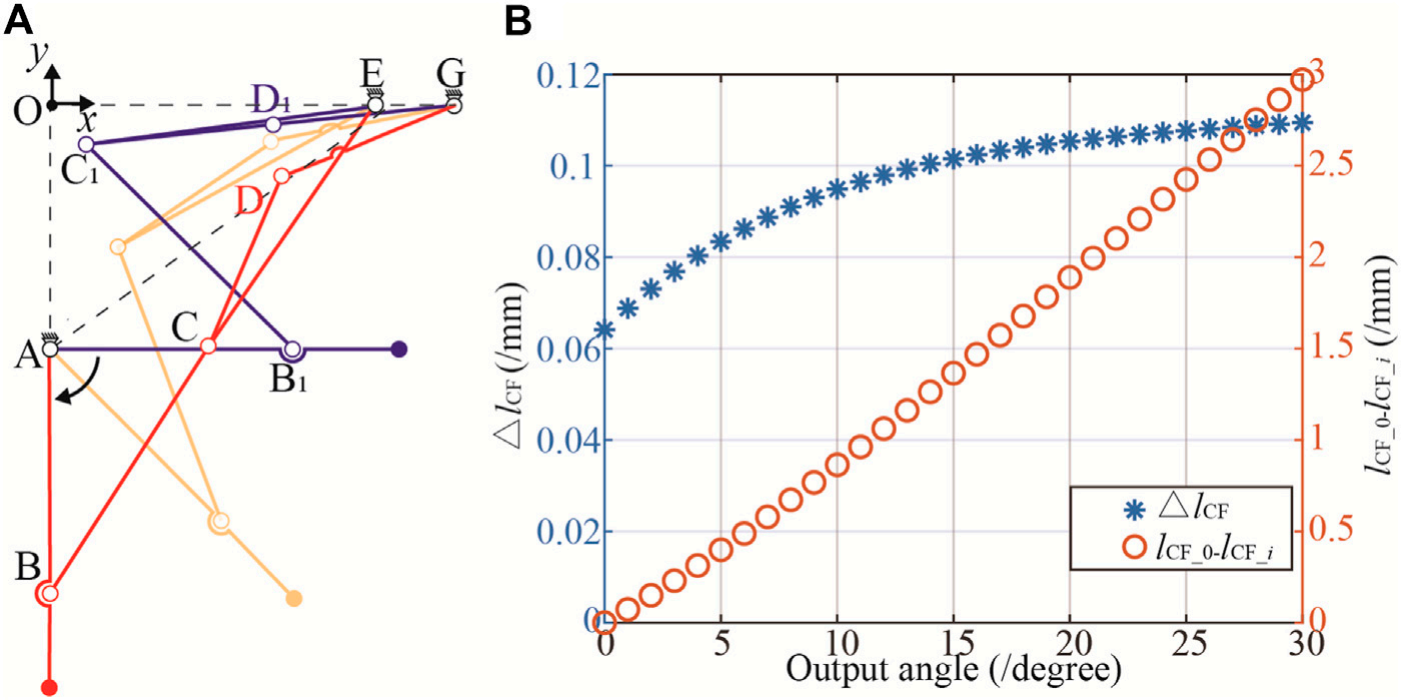
Simulation of the transformation process. **(A)** Motion trajectories of the mechanism. **(B)** The change in length of △*l*_CF_.

Upon substituting the structural parameters into Eqs 3 to 6, and setting the input/driving angles *θ*_1_ from 90-∠OAE to *θ*_1_ = 180-∠OAE, the motion trajectory of the mechanism transitioning from the retracted to the extended position is depicted in Figure 4A. Additionally, the variation in the length of C_1_F is illustrated in Figure 4B.

Figure 4A reveals that, within the two operational configurations of the Watt II six-bar linkage mechanism, the positional disparity between points D and D_1_ amounts to 34.1 mm. However, the actuation of this six-bar mechanism for positional switching is facilitated by the application of driving force at point D. The displacement of point D in these configurations requires the integration of a position compensation mechanism into the force application system, consequently elevating the structural complexity and compromising the stability of the device.

The collinearity of links CD and DG, i.e., *l*_CD_ + *l*_DG_ = *l*_C1G_, leads to the DoF of the Watt II six-bar linkage is 0 (F = 3N−2F_L_ = 3 × 4–2 × 6 = 0), consequently fixing the mechanism in a stationary state. Figure 4B illustrates the relationship between the angle of the output bar AB and the length of *l*_C1G_ when the mechanism is fixed. The variation in *l*_C1G_ per degree is remarkably small, less than 0.1 mm before reaching 15°. The cumulative length change, defined as the difference in *l*_C1G_ 's length at an output angle of 0° and at an angle *i* (*l*_CG_0_- *l*_CG_*i*_ ), has a maximum value of 2.97 mm, averaging 0.099 mm per degree. Such minute variations pose challenges in ensuring that AB remains in the required horizontal position during practical applications. The existing initial design demands high precision in the machining and assembly of the device. However, the operational environment of the device is often suboptimal, characterized by factors such as collisions and humidity, which lead to part deformation and rust. Consequently, precision cannot be guaranteed, preventing the wheel legs from locking in a horizontal position and causing wobble, which ultimately reduces the overall reliability of the device.

This paper addresses the identified deficiencies of the mechanism with its initial dimensions by optimizing the structural parameters through a multivariate constrained nonlinear optimization method (Scales, 1985). The primary objective of this optimization is to maintain the consistency of point D’s position in both the extended and retracted position. Consequently, the objective function is formulated as follows:

$$f{\left(x\right)}_{\min}={\left(x_{D_{1}}-x_{D}\right)}^{2}+{\left(y_{D_{1}}-y_{D}\right)}^{2}$$ (7)

where x = [ *l*_OA_, *l*_OE_, *l*_EG_, *l*_GF_, *l*_1_, *l*_2_, *l*_5_], (*x*_D_, *y*_D_) is the position of point D in the extended position of the mechanism and (*x*_D1_, *y*_D1_) is the position of point D in the retracted position of the mechanism.

The two positions of the reconfigurable mechanism are realised by BC-CE and C_1_D_1_-D_1_F covariance respectively, i.e., their constraints can be expressed as follows:

$$g_{1}\left(x\right)=l_{2}+l_{3}-l_{BE}=0\text{ if}\left(\text{AB}//y-\text{axis}\right)$$ (8)

$$g_{2}\left(x\right)=l_{5}+l_{6}-{l_{C_{1}}}_{F}=0\text{ if}\left(\text{AB }//\;x-\text{axis}\right)$$ (9)

where *l*_BE_ and *l*_C1F_ are derived by extrapolation from the kinematic model of the mechanism.

In order to avoid interference in the mechanism during motion, point C must never be co-linear with OA and OE in the motion of the mechanism, the expression for which is:

$$g_{3}\left(x\right)=x_{O}–x_{C_{1}}\leq 0$$ (10)

$$g_{4}\left(x\right)=x_{C_{1}}–x_{E}\leq 0$$ (11)

$$g_{5}\left(x\right)=y_{C_{1}}–y_{O}\leq 0$$ (12)

$$g_{6}\left(x\right)=y_{A}-y_{C_{1}}\leq 0$$ (13)

Applying the cosine theorem, extending GF’s length enhances the variation in C_1_F’s length during motion, thereby resolving the issue of minimal C_1_F length change that leads to mechanism instability. The design adheres to the criteria if C_1_F’s length variation exceeds 0.2 mm per 1° rotation of the AB, as informed by engineering expertise. This constraint is articulated as follows:

$$g_{7}\left(x\right)=0.2-l_{\text{CF}\_i}+l_{\text{CF}\_i-1}\leq 0\;\left(0<i\leq 90\right)$$ (14)

Finally, the structural parameter constraints of the linkage mentioned in Section 3.1 can be expressed as:

$$g_{8}\left(x\right)=l_{B_{1}E}-\left|l_{3}-l_{2}\right|\leq 0$$ (15)

$$g_{9}\left(x\right)=l_{C_{1}F}-\left|l_{5}-l_{6}\right|\leq 0$$ (16)

**FIGURE 5:**
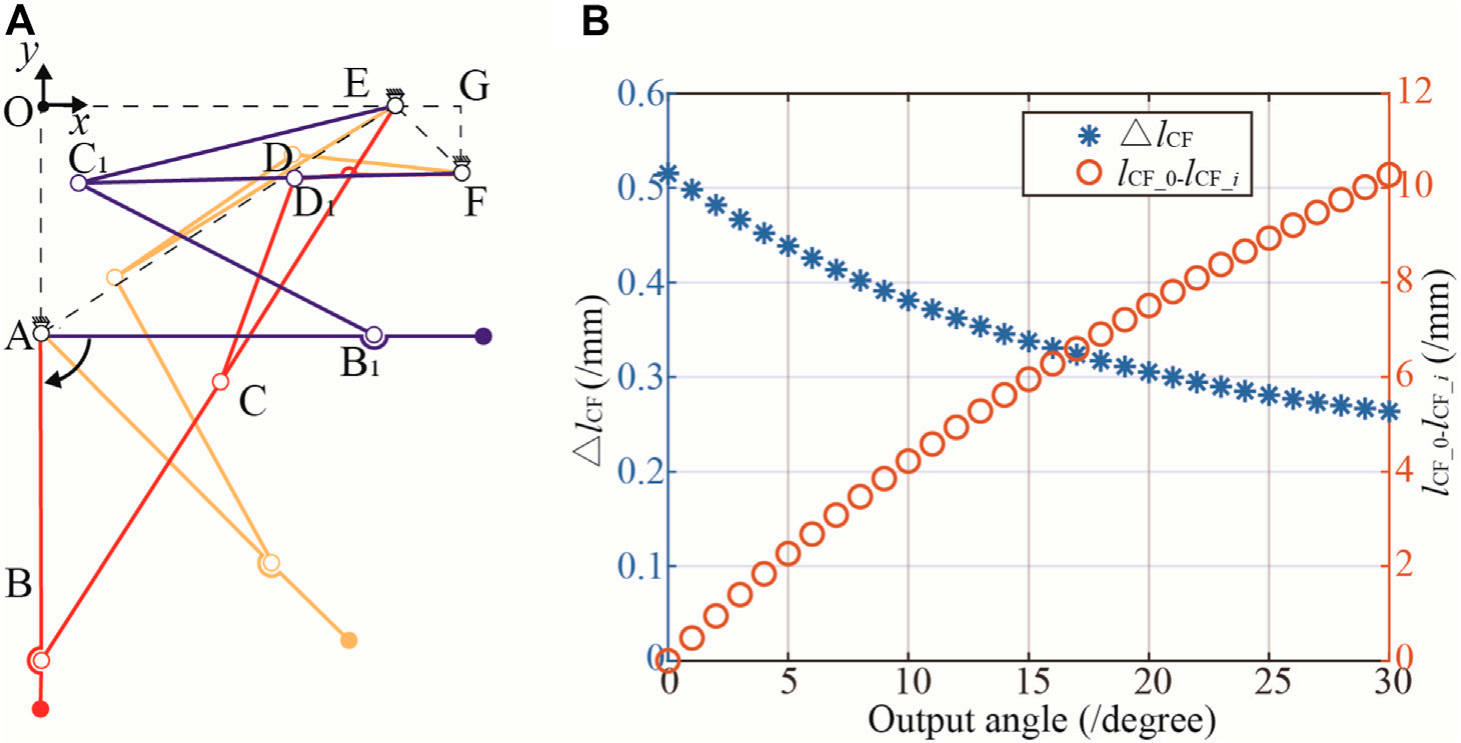
Optimized trajectories and △*l*_CF_. **(A)** Imoproved motion trajectories. **(B)** Optimized result.

Based on the constraints from Eq. 8 to Eq. 16, the objective function in Eq. 7 was solved, and the optimized structural parameters were obtained by rounding the results to one decimal place, as shown in Table 2. When these parameters are incorporated into the kinematic equations from Section 3; Figure 5A illustrates the extended and retracted positions of the reconfigurable frame. The positional deviation of point D is a minimal 2.3 mm, a discrepancy arising from the rounding to one decimal place. Such a deviation is deemed entirely acceptable for the design of wire-driven remote control systems. Figure 5B depicts the post-optimization variation in the length of C_1_F. The changes in C_1_F consistently exceed 0.2 mm/° and reach 0.5 mm when AB is at 0°, ensuring that the output link AB remains fixed in the horizontal position. The differential in *l*_CG___0_- *l*_CG___*i*_ attains a maximum of 10 mm, with an average variation of 0.3 mm/°. This level of precision allows the mechanism to maintain its fixed position even under deformation during use, thereby enhancing the equipment’s stability.

**TABLE 2:** Optimized structure parameters of the reconfigurable frame.

Structure parameter	*l*_OA_	*l*_OE_	*l*_EG_	*l*_GF_	*l*_1_	*l*_2_	*l*_3_	*l*_5_	*l*_6_
Length(mm)	137	210	40.1	40.1	197	199.1	195.6	130.1	100.2

## 5 Analysis of the driving force of the reconfigurable frame

**FIGURE 6:**
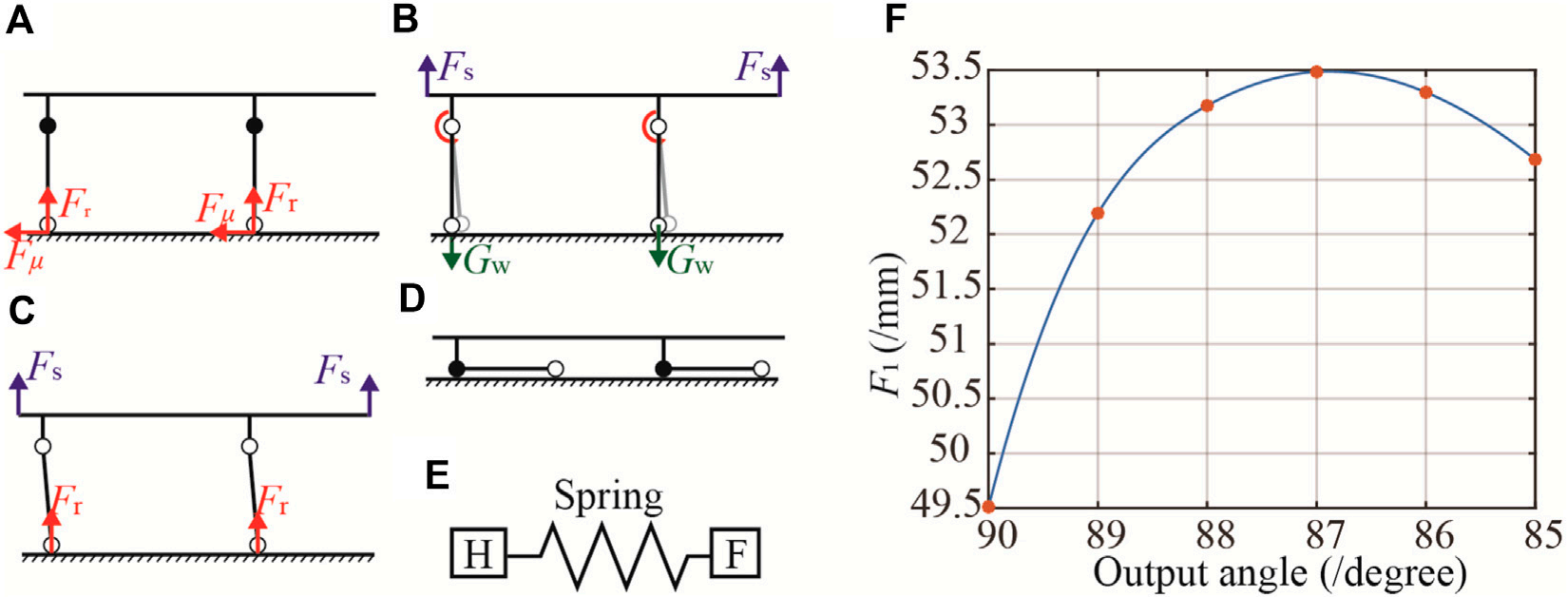
The transformation of stretcher vehicle mode to the stretcher mode. **(A)** Distribution of forces on aid in stretcher vehicle mode when bearing a casualty. **(B)** Depicts initial transition step with a 5° wheel leg rotation to increase the ground reaction lever arm. **(C)** Highlights smoother mode transition due to increased lever arm. **(D)** Finalizes the stretcher mode transition. **(E)** The elastic mechanism. **(F)** Force-displacement curve.

During stretcher vehicle operations for casualty transport, there is a risk of rescuers accidentally engaging the handbrake or other objects inadvertently triggering it, potentially leading to the unintended unlocking of the reconfigurable frame. Such occurrences could cause the wheel legs to fail and the stretcher vehicle to fall. To avert these situations, the protocol for transferring from the stretcher vehicle to the stretcher requires rescuers to stabilize the weight of the casualty and aid before engaging the handbrake to unlock the reconfigurable frame. Furthermore, an elastic mechanism has been incorporated into the drive system (Figure 6E) to constrain the maximum tensile force applied by the handbrake to point D of the reconfigurable frame, ensuring the frame remains locked even if the handbrake is activated before lifting the stretcher vehicle.

This section applies the principle of virtual work to determine the minimum actuation force required to disengage the locking mechanism at the dead center of the reconfigurable frame (extended and retracted states), thus determining the lower force threshold of the elastic mechanism. In addition, the drive force required to disengage the frame in the extended state during the transportation of a casualty is calculated, thus determining the upper force threshold.

The force-displacement properties of the Watt II six-bar linkage are modeled in this section using the virtual work principle. It should be noted that for simplicity purpose, the weight of the connecting rod is neglected in the modeling process and the torque of the torsion spring and the weight of the universal wheel are taken into account.

The principle of virtual work posits that for a multibody system in equilibrium under external forces, the total work done by these forces for any virtual velocity is zero. By introducing the generalized virtual velocities, denoted as δ*θ*, and acknowledging that the work from internal constraint reactions is null, the total virtual work, represented as δ*W*, can be expressed as follows:

$$\delta W=\vec{F_{1}}\delta \vec{Z_{1}}+M_{4}\cdot \delta \vec{\theta_{4}}+M_{6}\cdot \delta \vec{\theta_{6}}+\vec{F_{2}}\delta \vec{Z_{2}}=0$$ (17)

where *F*_1_ and *F*_2_ correspond to the tensile force and the force at the caster, respectively. *Z*_*i*_ represents the displacement vector associated with the force, *M*_*i*_ denotes the torque generated by the torsion spring, and *θ*_*i*_ is the angular displacement vector corresponding to the torque.

Using *θ*_6_ as the generalized coordinate, Eq. 17 can be derived:

$$\delta W=-F_{1}l_{6}\sin\left(\theta_{6}+\theta_{EFG}\right)\delta \theta_{6}+k_{6}\left(\theta_{6}-\theta_{60}\right)\delta \theta_{6}+k_{4}\left(\theta_{4}-\theta_{40}\right)\theta_{46}^{\prime }\delta \theta_{6}+F_{2}l_{1}\theta_{16}^{\prime }\delta \theta_{6}=0$$ (18)

where *k*_*i*_ is the torsional spring constant, and *θ*_*i* 0_ represents the angle of the torsion spring when undeformed. Solving Eq. 18 yields the linear driving force *F*_1_ as follows:

$$F_{1}=\frac{k_{1}\left(\theta_{6}-\theta_{60}\right)+k_{2}\left(\theta_{4}-\theta_{40}\right)\theta_{46}^{\prime }+F_{2}l\theta_{16}^{\prime }}{l_{6}\sin\left(\theta_{6}+\theta_{EFG}\right)}$$ (19)

where $\theta_{46}^{\prime }$ and $\theta_{16}^{\prime }$ are the linkage motion coefficients (Pennock and Israr, 2009), which will subsequently be determined through the linkage kinematics model.

The loop equations for the four-bar linkage ABCE and ECDF, when written as scalar equations, are as follows:

$$l_{1}\cos\left(\theta_{1}\right)+l_{2}\cos\left(\theta_{2}\right)-l_{3}\cos\left(\theta_{3}\right)-l_{4}\cos\left(180{}^{\circ}\right)=0$$ (20)

$$l_{1}\sin\left(\theta_{1}\right)+l_{2}\sin\left(\theta_{2}\right)-l_{3}\sin\left(\theta_{3}\right)-l_{4}\sin\left(180{}^{\circ}\right)=0$$ (21)

$$l_{3}\cos\left(\theta_{4}\right)+l_{5}\cos\left(\theta_{5}\right)-l_{6}\cos\left(\theta_{6}\right)-l_{7}\cos\left(180{}^{\circ}\right)=0$$ (22)

$$l_{3}\sin\left(\theta_{4}\right)+l_{5}\sin\left(\theta_{5}\right)-l_{6}\sin\left(\theta_{6}\right)-l_{7}\sin\left(180{}^{\circ}\right)=0$$ (23)

Given that $\theta_{3}=\theta_{4}+\theta_{OEA}+\theta_{GEF}=\theta_{4}+\theta_{t}$ , differentiation of Eqs 20, 21 with respect to *θ*_4_ yields:

$$l_{1}\sin\left(\theta_{1}\right)\theta_{14}^{\prime }+l_{2}\sin\left(\theta_{2}\right)\theta_{24}^{\prime }-l_{3}\sin\left(\theta_{4}+\theta_{t}\right)=0$$ (24)

$$l_{1}\cos\left(\theta_{1}\right)\theta_{14}^{\prime }+l_{2}\cos\left(\theta_{2}\right)\theta_{24}^{\prime }-l_{3}\cos\left(\theta_{4}+\theta_{t}\right)=0$$ (25)

Differentiating Eqs 22, 23 with respect to *θ*_6_ give:

$$l_{3}\sin\left(\theta_{4}\right)\theta_{46}^{\prime }+l_{5}\sin\left(\theta_{5}\right)\theta_{46}^{\prime }-l_{6}\sin\left(\theta_{6}\right)=0$$ (26)

$$l_{3}\cos\left(\theta_{4}\right)\theta_{46}^{\prime }+l_{5}\cos\left(\theta_{5}\right)\theta_{46}^{\prime }-l_{6}\cos\left(\theta_{6}\right)=0$$ (27)

Writing Eqs 24, 25 in matrix form gives:

$$\left[\begin{array}{cc} l_{1}\sin\left(\theta_{1}\right) & l_{2}\sin\left(\theta_{2}\right)\\ l_{1}\cos\left(\theta_{1}\right) & l_{2}\cos\left(\theta_{2}\right) \end{array}\right]\left[\begin{array}{c} \theta_{14}^{\prime }\\ \theta_{24}^{\prime } \end{array}\right]=\left[\begin{array}{c} l_{3}\sin\left(\theta_{4}+\theta_{t}\right)\\ l_{3}\cos\left(\theta_{4}+\theta_{t}\right) \end{array}\right]$$ (28)

and writing Eqs 26, 27 in matrix form gives:

$$\left[\begin{array}{cc} l_{3}\sin\left(\theta_{4}\right) & l_{5}\sin\left(\theta_{5}\right)\\ l_{3}\cos\left(\theta_{4}\right) & l_{5}\cos\left(\theta_{5}\right) \end{array}\right]\left[\begin{array}{c} \theta_{46}^{\prime }\\ \theta_{56}^{\prime } \end{array}\right]=\left[\begin{array}{c} l_{6}\sin\left(\theta_{6}\right)\\ l_{6}\cos\left(\theta_{6}\right) \end{array}\right]$$ (29)

Using Cramer’s rule (Wang and Sun, 2004), the first-order kinematic coefficients can be written from Eqs 28, 29 as

$$\theta_{14}^{\prime }=\frac{l_{3}\sin\left(\theta_{4}+\theta_{t}-\theta_{2}\right)}{l_{1}\sin\left(\theta_{1}-\theta_{2}\right)}$$ (30)

$$\theta_{46}^{\prime }=\frac{l_{6}\sin\left(\theta_{6}-\theta_{5}\right)}{l_{3}\sin\left(\theta_{4}-\theta_{5}\right)}$$ (31)

$$\theta_{16}^{\prime }=\theta_{14}^{\prime }\times \theta_{46}^{\prime }=\frac{l_{3}\sin\left(\theta_{4}+\theta_{t}-\theta_{2}\right)}{l_{1}\sin\left(\theta_{1}-\theta_{2}\right)}\times \frac{l_{6}\sin\left(\theta_{6}-\theta_{5}\right)}{l_{3}\sin\left(\theta_{4}-\theta_{5}\right)}=\frac{l_{6}\sin\left(\theta_{4}+\theta_{t}-\theta_{2}\right)\sin\left(\theta_{6}-\theta_{5}\right)}{l_{1}\sin\left(\theta_{1}-\theta_{2}\right)\sin\left(\theta_{4}-\theta_{5}\right)}$$ (32)

Substitution of Eq. 30 and Eq. 32 into Eq. 19 yields the resultant expression:

$$F_{1}=\frac{k_{1}\left(\theta_{6}-\theta_{60}\right)+k_{2}\left(\theta_{4}-\theta_{40}\right)\frac{l_{6}\sin\left(\theta_{6}-\theta_{5}\right)}{l_{3}\sin\left(\theta_{4}-\theta_{5}\right)}+F_{2}l\frac{l_{6}\sin\left(\theta_{4}+\theta_{t}-\theta_{2}\right)\sin\left(\theta_{6}-\theta_{5}\right)}{l_{1}\sin\left(\theta_{1}-\theta_{2}\right)\sin\left(\theta_{4}-\theta_{5}\right)}}{l_{6}\sin\left(\theta_{6}+\theta_{EFG}\right)}$$ (33)

In the stretcher vehicle mode, when bearing a casualty, the force distribution on the aid is illustrated in Figure 6A. The casters experience a reactive force from the ground, generating friction that impedes movement when the reconfigurable framework is unlocked, described by the equation *F*_2_ *l* = *F*_*μ*_×*l*_AW_ in Eq. 33. From this, *F*_1_ is determined to be 107.6 N. To transition from the stretcher vehicle mode to stretcher mode (Figure 6D), the reconfigurable framework is unlocked using a handbrake, and additional force is applied to rotate the wheel leg by 5°, as shown in Figure 6B, thereby increasing the lever arm of the ground reaction force and facilitating a smoother mode transition, as depicted in Figure 6C. Under this method, in Eq. 33, *F*_2_ *l* = G_W_×sin(*θ*_1_ + *∠OAE*)×*l*_AW_, and the variation in *F*_1_ is presented in Figure 6F. The maximum force and force-displacement curves obtained from the above can be used as the basis for the design of the spring mechanism.

## 6 Prototype development and field tests

**FIGURE 7:**
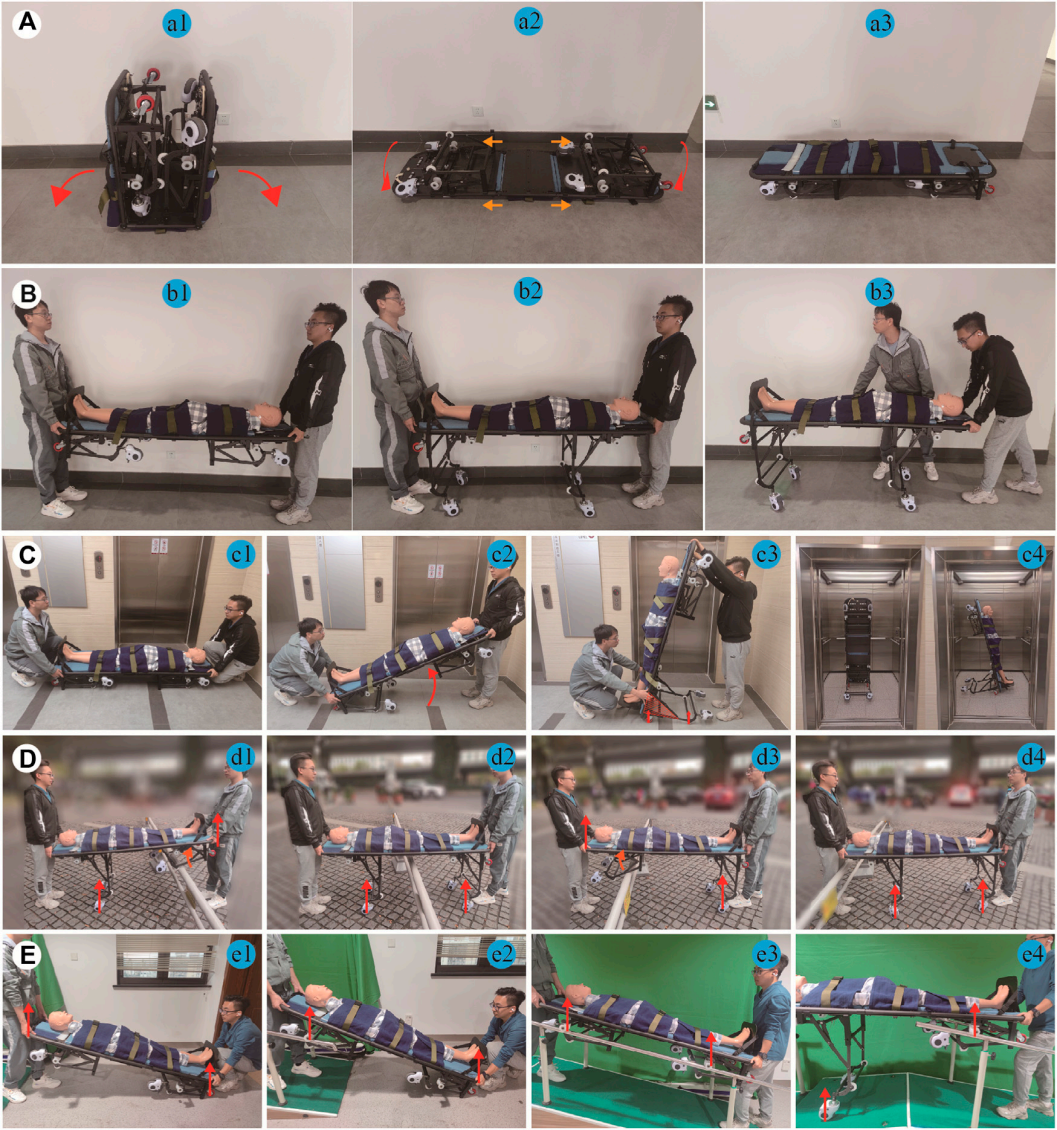
RMTACTA’s various locomotion modes. **(A)** Procedure for switching from folding mode to stretcher mode. **(B)** Procedure for switching from stretcher mode to stretcher vehicle mode. **(C)** Procedure for switching from stretcher mode to upright mode. **(D)** Demonstration of the workflow of the aid in obstacle-crossing mode. **(E)** Demonstration of the workflow of the aid in gangway-passing mode.

Based on the aforementioned theoretical analysis, a physical prototype of the proposed RMTACTA is developed using the structural characteristics obtained in Table 2 with some necessary modifications to the mechanical component design, as shown in Figure 7. It consists of two pairs of reconfigurable frames, two sets of telescopic gangway adaptor modules, a casualty immobilization module, and a pair of tilt-support gimbals. The aid can transition into six motion modes: folding mode (a1), stretcher mode (b1), stretcher vehicle mode (b2), upright mode (c), obstacle-crossing mode (d), and gangway-passing mode (e).

The equipment’s structural framework is constructed from carbon fiber tubing and panels; connectors are precision-engineered from aluminum alloy through CNC machining and undergo surface oxidation treatment; handrails are produced from nylon via 3D printing techniques. This results in a low production cost for the equipment, which maintains a lightweight profile at merely 20 kg.

Field tests are conducted on the physical prototype to evaluate and verify the performance of the proposed casualty transport aid. These tests utilized a medical mannequin to replicate a casualty, and involved two rescuers. Each rescuer can realize the switching of multiple modes of the casualty transport aid by controlling the handbrake on one side separately.

The transport aid is initially mode in the folding mode for expedited delivery to emergency locations, and it can be quickly converted into the stretcher mode, as shown in Figure 7 a1 to Figure 7 a3. The conversion process entails laying the aid‘s front and rear ends flat on the ground, shifting the sleeve to secure the hinge, and then inverting the aid. This stretcher mode enables rescuers to effortlessly lift casualties from the ground onto the stretcher for immediate first aid, providing an advantage during short-range transport, as demonstrated in Figure 7 a3. In flat terrain, as shown in Figure 7 b3, the rescuer can convert the aid to stretcher mode by applying the handbrake and lifting the stretcher, thus increasing the efficiency of the transfer, reducing the turning radius, and decreasing the physical burden on the rescuer.

When needing to enter or exit elevators or navigate tight corners, the rescuer can transition the transport aid from stretcher mode to upright mode. Specifically, the rescuer at the device’s rear operates the handbrake to unlocking the locked reconfigurable frame, and the rescuer at the device’s head raises it to an 80° angle with the ground. Meanwhile, the reconfigurable frame at the rear locks in the extended mode automatically, forming a stable mobile platform with the wheels on the wheel legs and the title-support gimbal, as depicted in Figure 7C. In upright mode, the rescuer can easily maneuver the transport aid, entering and exiting elevators and navigating tight corners.

When the transport aid encounters obstacles such as high thresholds, rescuers utilize a handbrake to unlock the reconfigurable framework, enabling the wheel-legs to rotate upon encountering a reactive force from the obstacle, thus allowing the equipment to pass the obstacle. During this process of passing obstacles, a set of support wheels maintains continuous contact with the ground, thereby reducing the physical strain on rescuer and ensuring the stability of the transport aid, as illustrated in Figure 7D.

When encountering a gangway, rescue personnel can facilitate the passage of the equipment along with the injured by dragging, rather than lifting, through the gangway. This is achieved by engaging the pulleys of the telescopic gangway adaptor module with the handrail of the gangway, as illustrated in Figure 7E. To ensure safety, this experiment was conducted using an indoor staircase instead of a gangway. Furthermore, during the passage, the aid ensures that at least one set of wheels is in contact with the ground or the gangway, thereby assisting rescue personnel in sharing the burden of the casualty’s weight.

The tests conducted in this study not only validate the structural design, mathematical model, and optimization approach but also demonstrate the effectiveness of the transfer equipment developed according to the proposed design principles, which exhibits exceptional capability in casualty transfer. When using this equipment, rescuers can use a simpler and safer method for transferring casualties. Subsequent testing of the aid’s casualty transportation capabilities will be conducted in a real-world industrial environment.

## 7 Conclusion

This paper presents the inaugural development of a reconfigurable multi-terrain adaptive casualty transport aid (RMTACTA) for emergency rescue within industrial settings. The device boasts six distinct operational modes: stretcher, stretcher vehicle, folding, gangway-passing, obstacle-crossing, and upright. Reconfigurability of the proposed aid is achieved by the creative design through the combination of a reconfigurable Watt II six-bar linkage framework with various functional modules.

Mechanical design for a casualty transport aid was presented and kinematic and static analysis of the reconfigurable framework. Optimization of the framework’s structural parameters was achieved through an investigation into the actuation points’ spatial positioning and the framework’s fixed orientation conditions. Employing the principle of virtual work, a mechanical analysis was performed on the optimized structural parameters of the reconfigurable framework to establish the upper and lower limits of the driving force required for the mechanism’s state transitions. These limits serve as the criteria for designing the elastic elements of the line-driven mechanisms.

The physical prototype of the RMTACTA was developed based on optimized structural parameters and mechanical analysis. Subsequent field trials were conducted to demonstrate the design concept, feasibility, and human-machine interaction of the proposed transport aid. The test results show that the RMTACTA can be reconfigured to meet various terrain and functional requirements. Additionally, the majority of mode changes can be controlled by rescue personnel using a single handbrake, allowing them to keep their hands on the aid’s handles throughout the casualty transport process, significantly reducing the operational effort required.

The novel proposed reconfigurable casualty transportation aid has provided novel insights into the design of robots for emergency medical transport and the improvement of casualty evacuation methods, which has potential applications in earthquake relief, maritime rescue, and battlefield medical support.

## Data Availability

The original contributions presented in the study are included in the article/Supplementary Material, further inquiries can be directed to the corresponding authors.
